# Quantification of malaria parasite release from infected erythrocytes: inhibition by protein-free media

**DOI:** 10.1186/1475-2875-6-61

**Published:** 2007-05-21

**Authors:** Svetlana Glushakova, Dan Yin, Nicole Gartner, Joshua Zimmerberg

**Affiliations:** 1 Laboratory of Cellular and Molecular Biophysics, National Institute of Child Health and Human Development, National Institutes of Health, Bethesda, MD 20892, USA

## Abstract

**Background:**

Intracellular malaria parasites leave their host erythrocytes to infect neighbouring cells after each cycle of asexual replication. No method is currently available for the direct quantification of parasite release.

**Method and results:**

To quantify parasite release process, human erythrocytes infected with *Plasmodium falciparum *were injected into sealed chambers at optimal density, where they progressed through the end of the erythrocyte cycle. Each event of parasite release inside the chamber at the site of erythrocyte rupture leaves on the chamber wall a footprint, composed of 1) separated parasites, 2) a digestive vacuole with haemozoin, and 3) fragments of the ruptured membranes. These footprints are stable for hours, allowing precise identification using differential interference contrast (DIC) microscopy. The relative rate of parasite release is defined as the percent of such footprints out of all schizonts injected and incubated into chamber at 37°C for two hours. The method is highly reproducible, easy to perform, and does not require expensive equipment. Additionally, this method allows one to analyse cell and release site morphology, which adds information about the release process and the quality of the culture. The method is used here to show that swelling of schizonts caused by protein-free media inhibits parasite release.

**Conclusion:**

In this study, a novel method is described to count sites of parasite release by microscopy. Besides the direct estimation of parasite release from infected erythrocytes, this method provides a morphological evaluation of normal infected cells approaching the end of the plasmodial life cycle, or pathological forms accumulated as the result of experimental intervention in the parasite release process. One may now accurately estimate the relative parasite release rate at the time of cycle transition, without any obligatory coupling to parasite invasion.

## Background

The asexual erythrocyte cycle of the human malaria parasite *Plasmodium falciparum *causes severe forms of disease [1]. Invasion of an individual parasite into a red blood cell initiates the cycle; approximately 48 hours later release of 16 – 32 daughter parasites terminates the cycle to spread the infection. These two space-time coupled events, parasite release and invasion, termed cycle transition, is the shortest stage of the plasmodial erythrocyte cycle. Parasite release *in vitro *takes only several seconds [2], and parasite invasion is complete within the next few minutes [3]. Traditional microscopic evaluation of plasmodial maturation by using blood smears allows one to follow plasmodial cycle progression in infected cultures. Thus, an increased proportion of the ring stage-infected erythrocytes with higher parasitaemia in synchronized cultures indicate the end of the previous cycle and the beginning of the new one. The quantification of parasite release without any contribution of parasite invasion is the prerequisite for studying parasite release from host cells, a largely unexplored aspect of parasite biology.

## Materials and methods

*P. falciparum *strain 3D7 (ATCC, Manassas, VA) was grown in group A^+ ^or O erythrocytes in RPMI 1640 medium (Gibco) supplemented with 25 mM Hepes (Gibco), 4.5 mg/mL glucose (Sigma), 0.1 mM hypoxanthine (Gibco), 25 μg/mL gentamicin (Gibco) and 0.5% AlbuMax (Gibco) according to the Trager-Jensen method [4]. Parasitaemia was evaluated by counting 1,000 total cells in cultures labelled with acridine orange to detect parasitized cells (Molecular Probes, Eugene, OR). DIC microscopy was performed using an inverted light microscope (LSM 510, Zeiss) with a 100× oil objective, 1.4 NA, and a high NA condenser. Synchronization of cultures was performed by a combination of Percoll-enrichment and sorbitol-treatment procedures. Briefly, infected cultures were washed by centrifugation (400 × g, 5 min at RT), resuspended in warm AlbuMax-RPMI medium, and applied on the top of an equal volume of 65% solution of warm Percoll (Sigma) in PBS supplemented with 0.5% AlbuMax. After centrifugation (1500 × g, 15 min at RT) late-stage infected erythrocytes were collected from the medium-Percoll interface, washed twice in warm AlbuMax-RPMI medium by centrifugation (400 × g, 3 min at RT), and mixed with normal erythrocytes at ~1:2 ratio and final haematocrit ~1%. Cultures were incubated for four hours at 37°C, and the medium was changed at least once during this time. Transmission of infection was terminated by treating infected cells with 5% sorbitol [5], washing twice by centrifugation (400 × g, 4 minutes), and finally maintaining the culture at 0.1%–0.2% haematocrit for two days with frequent medium change.

## Results

### Assay description

**Figure 1:**
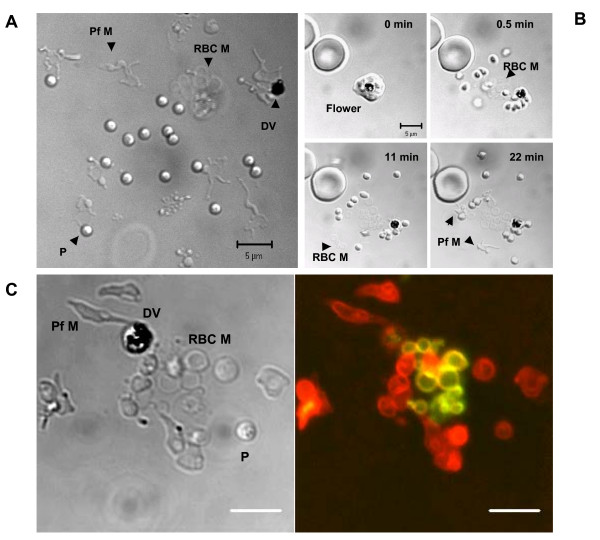
**Site of parasite release and its formation in the chamber. DIC light microscopy (A, B), DIC and fluorescent microscopy (C)**. **A**. A representative site. P – parasite, DV – digestive vacuole with haemozoin, Pf M – fragments of the parasite-derived membranes, RBC M – ruptured RBC membrane. **B**. Formation of the site in the chamber. Set of four timed pictures for a parasite releasing erythrocyte. Note that RBC- and parasite-derived membrane fragments are better detected at the extended time post parasite release. Flower – a pre-release stage of a schizont inside an infected RBC [2]. **C**. Identification of the membrane fragments' origin using differentially labelled infected RBC as described elsewhere [2]. Red colour objects – parasites (P) or fragments of parasite-derived internal membranes (Pf M) labelled with FM 4-64. Yellowish-green objects – blebs and vesicles originated from the ruptured RBC membrane (RBC M) labelled with FM 4-64 (red colour) and streptavidin-QD (green colour). Scale bar equals 5 μm.

The unexpected finding of preserved "sites of parasite release" from ruptured infected erythrocytes in sealed chambers filled with synchronized cultures of *P. falciparum *undergoing cycle transition [2] is the basis of this method. Sites of parasite release (Figure 1A–C) are composed of 1) fragments of infected erythrocyte membranes, 2) a few parasites, and 3) a haemozoin-containing digestive vacuole. All three elements are found situated together in a limited space of 663 ± 22 μm^2 ^(mean ± S.D., 11 sites in 3 independent experiments), presumably at the sites where infected erythrocytes rupture. The formation of release sites in their final form takes approximately 20–30 minutes. The light membrane fragments released at the moment of erythrocyte rupture initially float and become visible only after precipitating onto the chamber wall in the vicinity of the actual site of parasite release (Figure 1B). These sites can be easily detected and counted without additional labelling of cells using a light microscope (preferably DIC microscopy).

**Figure 2:**
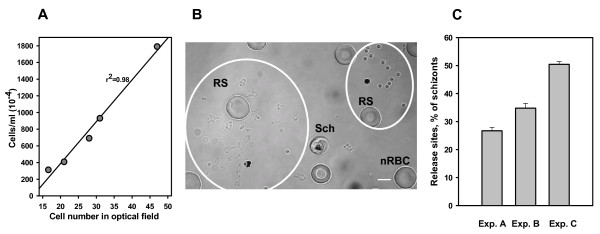
**Relative rates of parasite release in synchronized cultures of *P. falciparum *at the optimal cell density in chamber**. **A**. Range of cell densities in chambers suited for the release assay. Cells were injected into the chambers used for the release assay and cell number per optical field of 100× objective was evaluated as mean of 10 counts. Cell concentrations in the same cell suspensions were evaluated using Cellometer (Nexcelom Bioscience, Lawrence, MA). **B**. Optical field with two non-overlapping release sites (RS), normal RBC (nRBC) and one schizont (Sch). DIC light microscopy. Scale bar equals 5 μm. **C**. Representative experiments (three of twelve) demonstrating the range of parasite release in different cultures. Mean ± s.e. of data from three chambers, 300 counted cells per chamber.

For the identification of the compartmental origin of any particular membrane fragment, one must use differentially labelled infected cells (preparation described elsewhere [2]). Briefly, non-specific labelling of infected cells with membrane dye FM4-64 supplemented with the differential labelling of the biotinylated erythrocytes membranes with streptavidin-Quantum Dots allows one to identify FM 4-64-labeled parasite-derived membranes (red colour fragments, Figure 1C) and double-labelled erythrocyte membranes (yellowish-green colour fragments, Figure 1C). A detailed morphological description of membrane fragments at the sites of parasite release is presented in our early publication [2]. The relative rate of release can be expressed as the fraction of ruptured infected erythrocytes out of all schizonts injected into a chamber and incubated for two hours at 37°C, assuming that one ruptured infected erythrocyte yields one site of release (Figure 2). The optimal protocol for the release assay is described below.

The release assay should be performed using highly synchronized parasite culture. In this study culture was synchronized using the Percoll enrichment/sorbitol treatment protocol [5]. Prompt procedural performance, moderate centrifugation forces (400 × g), and the use of warm (37°C) AlbuMax-containing Percoll or AlbuMax-containing RPMI 1640 medium for procedures allowed us to achieve the best result. It is necessary to use either freshly obtained or one to three days old normal human red blood cells (Type O or A+) as donor cells for initiation of synchronized cultures. For the transmission of infection from Percoll-isolated late stage infected cells to normal erythrocytes, cells were mixed in a 1:2 ratio and then incubated for four hours at 37°C at approximately 1% haematocrit. The medium was changed at least once during incubation period. The beginning of co-incubation of infected and normal erythrocytes served as time zero in the parasite life cycle. Following the incubation period, cultures were treated with 5% sorbitol to eliminate schizonts, washed twice with AlbuMax-containing medium, and then resuspended in the same medium at 0.1% – 0.2% haematocrit. The culture was then maintained for two days with frequent changes of medium.

**Figure 3:**
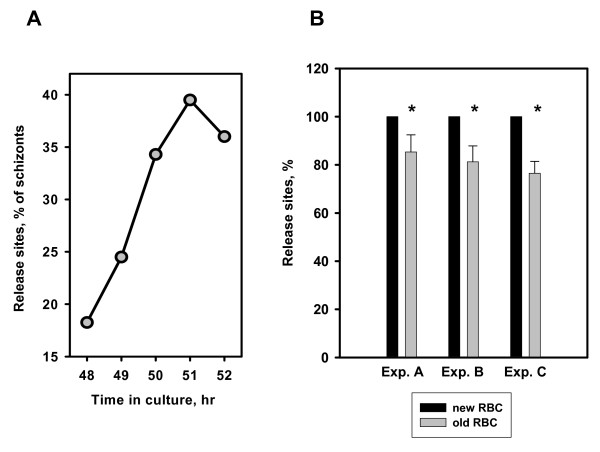
**Kinetics of parasite release in individual culture and effect of erythrocyte storage time on the relative rate of parasite release**. **A**. Synchronized culture of *P. falciparum *was taken for the evaluation of the relative rate of parasite release at the indicated time post-cultures initiation, and the assay was performed as described in the paper. Results presented as mean of 300 – 700 counted cells. **B**. The release assay was performed side-by-side with two synchronized cultures initiated simultaneously from the same infected cultures but in two different normal erythrocytes preparations with different storage age. Age of erythrocytes were: 3 and 12 days in Experiment A, 2 and 14 days in Experiment B and 0 and 19 days in Experiment C. Cultures were taken for the assay at 51 hr post cultures initiation. Result presented as mean of 300 – 500 counted cells ± s.e.

At the time of cycle transition, 49–51 hour-old cultures of cell suspensions at concentration ~2–18 × 10^6 ^and parasitaemia ~10% were injected into HBW20 chambers (Grace Bio-Labs, Bend, OR) under strict temperature control at 37°C. Chambers were then sealed for preservation of pH and gas composition optimal for parasite physiology. Loaded chambers were then incubated for two hours at 37°C in the dark to accumulate release sites and then rapidly cooled in the dark for one hour at 15°C on a thermo-plate to stop parasite release before microscopic examination at 20–22°C. Light microscopy (preferably DIC, but bright field is acceptable) was used for the quantification of the number of ruptured infected erythrocytes (estimated by the number of parasite release sites) that occurred in chambers during the incubation period. A high quality 100× oil objective, NA 1.4, and a high NA condenser are recommended for easier recognition of parasite release sites during the counting process. For optimal counting, the number of cells in the optical field of the objective should be in the range of 15–60 cells/field (Figure 2A). At these cell densities the release sites do not overlap each other and can be easily identified by the presence of a single digestive vacuole in the area covered by released parasites and membrane fragments (Figure 2B). High parasitaemia in infected cultures requires a lower cell density in chambers to avoid overlapping of release sites that may interfere with the quality of analysis. However, cell density in a chamber does not affect the relative rate of parasite release *per se *at the range indicated above (four experiments, data not presented). The peak of parasite release occurs between the 50^th ^and 52^nd ^hour post-initiation of synchronized cultures (Figure 3A) with a range of rates between 25% and 50% in different cultures (Figure 2C). Within this range of rates, release evaluation in one chamber (i.e. thrice counting 100 infected and ruptured cells) takes ~10 minutes. The main factors affecting the rate of parasite release are 1) the age of the erythrocytes used for preparation of synchronized cultures, and 2) the culture incubation time inside a chamber.

### Factors affecting parasite release

#### Kinetics of parasite release

A highly synchronized culture provides a predictable period of time during which most parasites undergo cycle transition (Figure 3A). We analysed the kinetics of parasite release from infected erythrocytes in three independent experiments that used blood (0 – 3 day old) from different donors. The highest rate of parasite release (41.25%, 39.5% and 49.4%; mean of 300–500 counted cells) was observed in the interval between the 50th and the 53rd hr from culture initiation. However, the kinetics of parasite release was different for each donor's blood and was affected by the length of time blood was stored (at 4°C) before culture initiation. "Old" blood reproducibly demonstrated lower parasite release rates relative to "fresh" blood in side-by-side experiments (Figure 3B). Consequently, one should only design experiments with parallel samples in the same donor blood; otherwise, kinetic curves need to be constructed for each new sample.

#### Time of culture incubation in chamber at 37°C

Since the actual rate of release is not linear throughout the cycle for synchronized cultures (Figure 3A), it is critical to determine the optimal chamber incubation period; two hours proved to be optimal in our hands. This time is sufficient for the rupture of up to 50% of the schizonts in 50–51 hr-old cultures; cultures do not deteriorate during this interval of time and the sites of parasite release are fully formed. Extension of incubation time from two to four hours significantly increased release numbers, but not relative rates (40% extent, ~10%/hr vs. 30.0% extent, 15%/hr, p = 0.02 in one experiment and 46% extent, 12 %/hr vs. 33% extent, 17%/hr, p < 0.01 in another experiment). However, morphologically damaged schizonts were observed in cultures with high parasitaemia (around 20%) after the four-hour incubation period at 37°C, indicating a deterioration of optimal conditions for parasite viability. To stop cycle transition at the end of the 37°C incubation period, the cooling of the chambers was varied. A set of experiments with multiple chambers showed no statistically significant differences between the results of "hot" (without cooling the chambers) and "cold" (with cooling the chambers) counts for the same cultures (n = 3, data not presented). However, data variability expressed as a percentage of the mean value increased significantly for the "hot" count protocol in comparison with the "cold" protocol (5.98 ± 0.41% vs. 2.98 ± 0.46%, p = 0.002, n = 3–4). Thus, cooling chambers to approximately 15–18°C for one hour before counting release sites and schizonts is recommended.

#### Medium selection for the release assay

**Figure 4:**
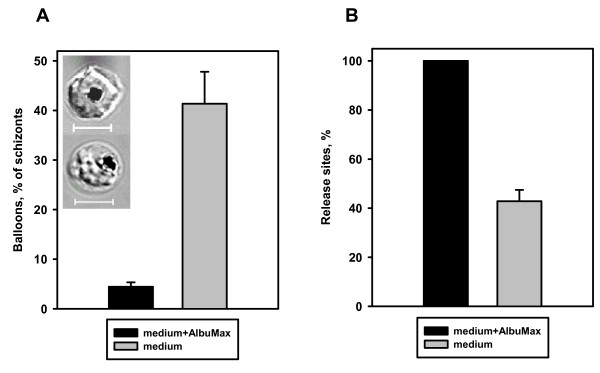
**Schizont swelling in protein-free medium inhibits parasite release**. **A**. Synchronized culture of *P. falciparum *was placed in different compositions of RPMI medium (with or without 0.5% AlbuMax) and immediately injected into the chambers for the release assay. The release assay was performed as described. Result presented as mean of 300 counted cells ± s.e. n = 6–8 experiments. Inset: DIC images of normal (upper) and swelled, ballooned, (lower) schizonts. Scale bar equals 5 μm. **B**. Cells were prepared as described in Figure 4A and relative rates of parasite releases were evaluated. Data presented as mean of 300 counted cells ± s.e. n = 5 experiments.

While developing the release assay, the observation was made that the normal parasite release process occurs only in protein-containing (AlbuMax or human serum) medium but not in pure medium or PBS. In non-protein containing medium, schizonts swelled massively, frequently hemolysing (Figure 4A). Accumulation of dead, ballooned schizonts correlated with sharply declining numbers of normal release events (Figure 4B). The reasons for this effect are not clear to us, but it does demonstrate the importance of actually viewing cell morphology to fully evaluate release performance.

## Discussion

Release of malaria parasites from infected RBC at the end of the asexual erythrocyte cycle occurs approximately every two days, asynchronously in parasite culture and synchronously *in vivo*. It remains one of the most elusive processes of the parasite life cycle. There are several reasons for why it is so poorly studied. First, release is a very short-lasting event. Second, it is difficult to observe release *in vitro *directly under the microscope because schizonts are very sensitive to the culture conditions. Third, schizonts have low physical stability and high sensitivity towards different temperature, pH, osmotic pressure, and light conditions. The release assay described in this paper is a compilation of optimal parameters to minimize sources of potential errors to results interpretation. A quantitative assay for parasite release employs procedures and conditions that are not harmful for schizont viability. Despite the fact that this assay does not directly observe parasite release, the assay provides an accurate microscopic quantification of release events by counting the release footprint from each ruptured infected erythrocyte. These sites of parasite release are preserved for hours in the sealed chambers and have a very specific morphological pattern that is different from the morphology of deteriorated schizonts. Thus, only normal schizonts and normal release sites are counted in this assay, and the relative parasite release rate can be estimated by itself, without any obligatory coupling to parasite invasion.

## Authors' contributions

SG conceived the method, designed and performed the experiments, drafted the manuscript. DY participated in the design of the study and performed the experiments. NG participated in manuscript preparation. JZ participated in study design and coordination and drafted the manuscript. All authors read and approved the final manuscript.
